# The mir-767-105 cluster: a crucial factor related to the poor prognosis of hepatocellular carcinoma

**DOI:** 10.1186/s40364-020-0186-7

**Published:** 2020-02-13

**Authors:** Tao Rui, Siyi Xu, Shi Feng, Xueyou Zhang, Haitao Huang, Qi Ling

**Affiliations:** 1 Division of Hepatobiliary and Pancreatic Surgery, Department of Surgery, First Affiliated Hospital, School of Medicine, Zhejiang University, 79 QingChun Road, Hangzhou, 310003 China; 2 NHFPC Key Laboratory of Combined Multi-organ Transplantation, Hangzhou, China; 3 Key Laboratory of the Diagnosis and Treatment of Organ Transplantation,CAMS, Hangzhou, China; 4 Key Laboratory of Organ Transplantation, Hangzhou, Zhejiang Province China; 5 Department of Electrocardiographic and Cardiac Examination, Zhejiang Provincial People’s Hospital, Hangzhou, 310014 China

**Keywords:** Hepatocellular carcinoma, Mirna cluster, Prognosis, TCGA

## Abstract

MiRNAs have been widely reported as the therapeutic target for hepatocellular carcinoma (HCC). However, mirna clusters, as the more impressive tumor regulatory factors, have received little attention. By deeply digging the Cancer Genome Atlas (TCGA) database, we aimed to explore the vital mirna cluster that regulated the poor prognosis of HCC. The results showed that the upregulation of mirna cluster-767-105 in HCC was the most significant, compared with the non-tumor tissues. Besides, high expression of all three members of the cluster was positively correlated with poor prognosis of HCC and the resistance of sorafenib. Cox analysis proved that all the three mirnas were independent prognostic factors, while the mir-767 was the most compelling (HR value 8.388, 95%CI 2.524–27.897). The higher expression of the three-mirna signature also significantly indicated the worse prognosis. Through bioinformatics analysis, we screened their common potential target genes, which were highly correlated with tumor regulation. These results supported that the mirna cluster-767-105 promoted the poor outcome of HCC and could be a robust target for the therapy of HCC patients.

**To the Editor:**

Hepatocellular carcinoma (HCC) remains one of the major causes of cancer mortality in the world [1, 2]. Although the progress of multidisciplinary therapy and the advancement of pharmacological treatment have benefited a minority of patients, the poor prognosis of HCC still exists [3]. Therefore, further researching the molecular mechanisms and therapeutic targets of HCC is urgent. The function of tumor-related miRNAs on tumorigenesis and tumor progression is well known [4–6]. While, miRNA cluster, defined as a miRNAs family that transcribed from adjacent genome, always intensely targets the same pathway and achieve synergistic regulation activity [7, 8]. In this study, we focused on exploring one of the most valuable miRNA clusters of HCC, which was mined from The Cancer Genome Atlas (TCGA) database.

The mirnas profiles of 373 HCC samples and 50 Non-tumor samples were acquired from TCGA. Then the differentially expressed mirnas were screened out according to the fold-change (FC) values of expression levels. We surprisingly found that the three mirnas, with the top1–3 differentiated FC values, belong to the mirna cluster-767-105 (Fig.1.a and Additional file 1: Table S1). Then we extracted the expression data of each sample for accurate visual comparison. The Scatter plots (Fig.1.b) and the heatmap (Fig. 1.c) showed that the mir-767-105 cluster was significantly and consistently upregulated in HCC.

**Fig. 1:**
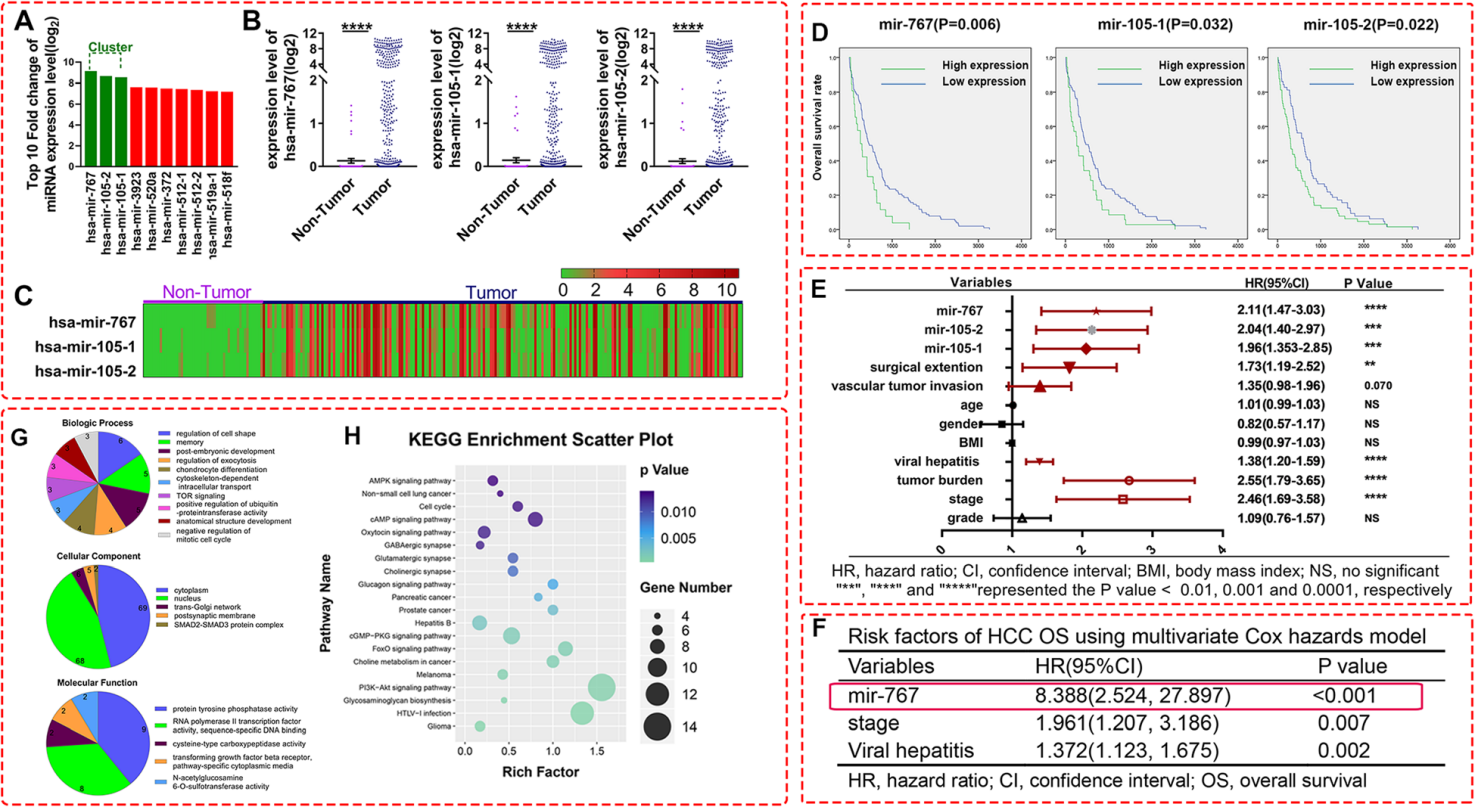
legend. TCGA database exploration of mir-767-105 cluster in hepatocellular carcinoma (HCC). **a** Top 10 mirna expression levels in HCC. The top 1–3 belonged to mir-767-105 cluster. **b** Comprehensive comparison of the mir-767-105 cluster expression levels between the HCC samples and non-tumour samples. Error bars represented the mean ± SEM. ****represent a *P* -value < 0.0001. *P*-value was calculated with the Mann-Whitney test. **c** Heatmap of mir-767-105 expression levels from 50 non-tumour samples and 373 HCC samples. **d** Kaplan-Meier survival analysis with log-rank test between the low and high expression of the mir-767(left), mir-105-1(middle), and mir-105a-2(right) expression levels was performed. **e-f** The results of univariate Cox analysis was shown with the forest plot, and the multivariate Cox analysis was shown as Table. **g** The items from Gene Ontology in biological process, cellular component and molecular function. **h** The top 20 items from Kyoto Encyclopedia of Genes and Genomes

Given that the dramatically high expression of the mir-767-105 cluster may indicate that they play the crucial role in HCC, we further evaluated the effect of the mir-767-105 cluster on the survival of HCC and the histopathologic characteristics. By evaluating the clinical information of 373 HCC samples with Kaplan-Meier analysis, we found that the high expression of all three members of the cluster was positively correlated with poor prognosis (Fig.1.d). Of note, the expression levels of the three mirnas were much higher in the sorafenib resistant group, which suggested the three mirnas negatively influence the therapeutic effect of sorafenib on HCC (Additional file 1: Figure S1). Cox proportional hazards regression analysis was used to assess the risk factors of HCC progress. Among those recipient characteristics correlated with the poor survival of HCC, viral hepatitis, tumor burden, tumor stage, vascular tumor invasion, surgical extension, level of mir-105-1, level of mir-105-2, level of mir-767, were significantly associated with HCC poor survival (Fig.1.e), by univariate Cox analysis. We further verified those three mirnas has no significant correlation with the other clinicopathological characteristics above by chi-square test, suggesting that the three mirnas were independent prognostic factors (Additional file 1: Table S2). Then the statistically significant factors were entered into multivariate Cox models. Compared with the other two independent prognostic factors (viral hepatitis, tumor stage), the HR (hazard ratio) value (8.388, 95%CI 2.524–27.897) of mir-767 was significantly higher, proving that mir-767 strongly regulated the poor prognosis of HCC (Fig.1.f). For further exploring the influence of the 3-mirna signature on the prognosis of HCC, we divided the HCC samples into two clusters according to the 3-mirna different expression levels (Additional file 1: Figure S2.A), by the “ConsensusClusterPlus” package of R software. Chi-square test confirmed that there was no significant correlation between the 3-mirna signature and other clinical factors, except the status of HCC patients (Additional file 1: Figure S2.B). Kaplan-Meier analysis displayed that the higher expression cluster significantly indicated the worse prognosis (Additional file 1: Figure S2.C).

We further analyzed the bio-information and the synergistic mechanism of the mir767–105 cluster, by exploring the target genes of these three miRNAs with the three online mirna-target gene database TargetScan, miRWalk, and miRDB (Additional file 1: Figure S3.A). The overlapped genes were screened by the “venny” package of R software. 378 genes may be the common targets of the mir-767-105 cluster (Additional file 1: Figure S3.B). The protein-protein interaction (PPI) network showed the possible interaction mode and key nodes of these target genes (Additional file 1: Figure S4). Gene Ontology (GO) analysis (in three domains: biological process, cellular component and molecular function) indicated that the functions of the target genes were associated with protein tyrosine phosphatase activity, transcription factor activity (Fig.1.g). A mount of target genes was enriched in cytoplasm and nucleus, suggesting that the cluster might act on transcriptional regulation. Of note, of the top 20 items from Kyoto Encyclopedia of Genes and Genomes (KEGG), 12 (60%) are tumor-related, 4 (20%) are Metabolic regulation-related (Fig.1.h). It strongly implied that the cluster was highly correlated with the regulation of tumorigenesis, and that may be achieved through influencing the metabolic process of tumor. In summary, the significant upregulation of the mir-767-105 cluster may stimulate the HCC tumorigenesis and promote the poor outcome of HCC. It can serve as a biomarker and target for prognosis and therapy of HCC patients.

## Supplementary information

**Additional file 1: Table.S1** The information of mir-767-105 cluster from miRBase. **Figure S1** comparison of the mir-105-1 (left), mir-105-2 (middle), mir-767 (right) expression levels between the sorafinib sensitive group and sorafinib resistant group. Error bars represented the mean ± standard error of the mean. “*”, “**”, “***“represent a P -value < 0.05, 0.01, 0.001, respectively. **Table.S2** The correlation between the three mirnas and the clinicopathological characteristics. **Figure S2 S2 A)** According to the 3-mirna differentiated expression levels, the HCC samples were divided into two clusters, by “ConsensusClusterPlus” package. **S2 B)** Characteristics of the two cluster were presented with heatmap. The cluster 2 integrated HCC samples with relatively higher mir-767-105 cluster expression. **S2 C)** Kaplan-Meier survival analysis showed that the cluster 2 with relatively higher mir-767-105 cluster expression achieved poorer outcome. **Figure S3 S3 A)** The predicted target genes of mir-767 and mir-105 from the miRDB (red colour), miRWalk (green colour), TargetScan (blue colour) databases were overlapped. **S3 B)** The mir-767 (green colour) and mir-105 (red colour) target genes were further overlapped. A total of 378 genes may be co-targeted by the cluster. **Figure S4 S4)** Protein-protein interaction analysis showed the target nodes with the highest confidence.

## Data Availability

All data of this study are included in the article and its supplementary information files.

## Abbreviations

FC: Fold-change
GO: Gene Ontology
HCC: Hepatocellular carcinoma
HR: Hazard ratio
KEGG: Kyoto Encyclopedia of Genes and Genomes
PPI: Protein-protein interaction
TCGA: The Cancer Genome Atlas
