# Supplementary material for: The mir-767-105 cluster: a crucial factor related to the poor prognosis of hepatocellular carcinoma
Source: Biomark Res. 2020 Feb 13;8:7. doi: 10.1186/s40364-020-0186-7 (PMC7020499; doi:10.1186/s40364-020-0186-7)
Supplement: Supplementary file 1 — Additional file 1: Table.S1 The information of mir-767-105 cluster from miRBase. Figure S1 comparison of the mir-105-1 (left), mir-105-2 (middle), mir-767 (right) expression levels between the sorafinib sensitive group and sorafinib resistant group. Error bars represented the mean ± standard error of the mean. “*”, “**”, “***“represent a P -value < 0.05, 0.01, 0.001, respectively. Table.S2 The correlation between the three mirnas and the clinicopathological characteristics. Figure S2 S2 A) According to the 3-mirna differentiated expression levels, the HCC samples were divided into two clusters, by “ConsensusClusterPlus” package. S2 B) Characteristics of the two cluster were presented with heatmap. The cluster 2 integrated HCC samples with relatively higher mir-767-105 cluster expression. S2 C) Kaplan-Meier survival analysis showed that the cluster 2 with relatively higher mir-767-105 cluster expression achieved poorer outcome. Figure S3 S3 A) The predicted target genes of mir-767 and mir-105 from the miRDB (red colour), miRWalk (green colour), TargetScan (blue colour) databases were overlapped. S3 B) The mir-767 (green colour) and mir-105 (red colour) target genes were further overlapped. A total of 378 genes may be co-targeted by the cluster. Figure S4 S4) Protein-protein interaction analysis showed the target nodes with the highest confidence. [file 40364_2020_186_MOESM1_ESM.docx]

**Table.S1**. The information of mir-767-105 cluster from miRBase

| Office Symbol | miRBase | Chromosome Location |
| --- | --- | --- |
| hsa-mir-105-1 | MI0000111 | chrX: 152392219-152392299 |
| hsa-mir-767 | MI0003763 | chrX: 152393421-152393529 |
| hsa-mir-105-2 | MI0000112 | chrX: 152394412-152394492 |

**Fig.S1**


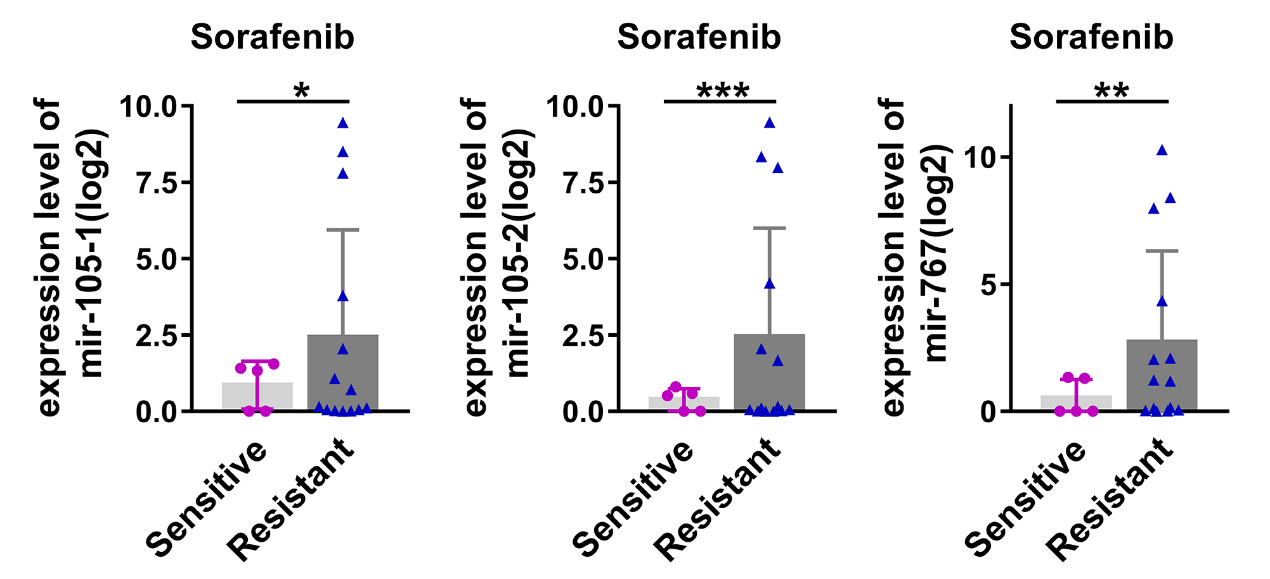


comparison of the mir-105-1 (left), mir-105-2 (middle), mir-767 (right) expression levels between the sorafinib sensitive group and sorafinib resistant group. Error bars represented the mean ± standard error of the mean. “*”, “**”, “***”represent a P -value < 0.05, 0.01, 0.001, respectively.

**Table.S2** The correlation between the three mirnas and the clinicopathological characteristics.

| Charactristics | has-mir-767(n) | | |  | Has-mir-105-1(n) | | |  | Has-mir-105-2(n) | | |
| --- | --- | --- | --- | --- | --- | --- | --- | --- | --- | --- | --- |
|  | Low | High | p |  | low | High | p |  | low | High | p |
| Age(y) |  |  |  |  |  |  |  |  |  |  |  |
| <65 | 191 | 41 | <0.001 |  | 130 | 102 | 0.004 |  | 129 | 103 | 0.008 |
| ≥65 | 89 | 49 |  |  | 56 | 82 |  |  | 57 | 81 |  |
| Gender |  |  |  |  |  |  |  |  |  |  |  |
| Female | 69 | 50 | 0.048 |  | 62 | 57 | 0.63 |  | 66 | 53 | 0.158 |
| Male | 117 | 135 |  |  | 124 | 128 |  |  | 120 | 132 |  |
| BMI(kg/m^2^ ) |  |  |  |  |  |  |  |  |  |  |  |
| ≥25 | 137 | 43 | 0.387 |  | 141 | 39 | 0.491 |  | 146 | 34 | 0.362 |
| <25 | 113 | 44 |  |  | 118 | 39 |  |  | 121 | 36 |  |
| Vascular_tumor |  |  |  |  |  |  |  |  |  |  |  |
| None | 156 | 50 | 0.448 |  | 107 | 99 | 0.983 |  | 163 | 43 | 0.994 |
| Invasion | 79 | 31 |  |  | 57 | 53 |  |  | 87 | 23 |  |
| Surgery extension |  |  |  |  |  |  |  |  |  |  |  |
| Segmentectomy | 136 | 39 | 0.171 |  | 138 | 37 | 0.258 |  | 142 | 33 | 0.374 |
| Lobectomy | 119 | 48 |  |  | 123 | 44 |  |  | 129 | 38 |  |
| Residual tumor |  |  |  |  |  |  |  |  |  |  |  |
| RO | 243 | 81 | 0.791 |  | 250 | 74 | 0.628 |  | 258 | 66 | 0.850 |
| R1+R2 | 13 | 5 |  |  | 13 | 5 |  |  | 14 | 4 |  |
| Viral hepatitis |  |  |  |  |  |  |  |  |  |  |  |
| Only HBV positive | 46 | 11 | 0.880 |  | 46 | 11 | 0.951 |  | 46 | 11 | 0.772 |
| Only HCV positive | 14 | 4 |  |  | 17 | 4 |  |  | 18 | 3 |  |
| HBV+HCV positive | 62 | 18 |  |  | 63 | 17 |  |  | 63 | 17 |  |
| Fibrosis(ishak score) |  |  |  |  |  |  |  |  |  |  |  |
| 0 | 56 | 19 | 0.863 |  | 58 | 17 | 0.788 |  | 58 | 17 | 0.622 |
| 1-4 | 44 | 16 |  |  | 46 | 14 |  |  | 48 | 12 |  |
| 5-6 | 61 | 18 |  |  | 64 | 15 |  |  | 66 | 13 |  |
| Tumor burden |  |  |  |  |  |  |  |  |  |  |  |
| Low | 202 | 73 | 0.641 |  | 208 | 67 | 0.780 |  | 218 | 57 | 0.307 |
| High | 66 | 27 |  |  | 69 | 24 |  |  | 69 | 24 |  |
| Tumor Stage |  |  |  |  |  |  |  |  |  |  |  |
| Low | 191 | 66 | 0.697 |  | 196 | 61 | 0.892 |  | 205 | 52 | 0.401 |
| High | 65 | 25 |  |  | 68 | 22 |  |  | 68 | 22 |  |
| Tumor grade |  |  |  |  |  |  |  |  |  |  |  |
| Low | 173 | 58 | 0.294 |  | 179 | 52 | 0.243 |  | 187 | 44 | 0.096 |
| High | 95 | 41 |  |  | 98 | 38 |  |  | 100 | 36 |  |
| **Event** |  |  |  |  |  |  |  |  |  |  |  |
| **Alive** | **190** | **53** | **0.002** |  | **194** | **49** | **0.007** |  | **201** | **42** | **0.003** |
| **Death** | **81** | **47** |  |  | **86** | **42** |  |  | **89** | **39** |  |

**Fig.S2**


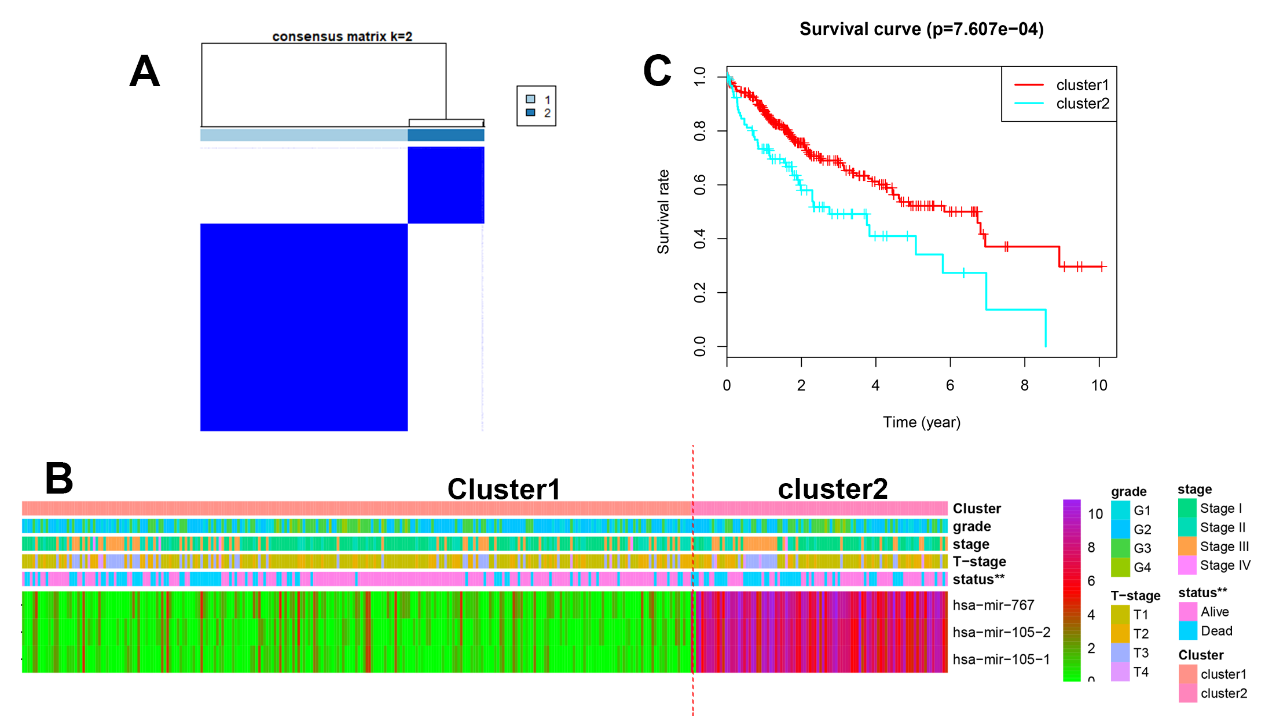


**S1 A)** According to the 3-mirna differentiated expression levels, the HCC samples were divided into two clusters, by “ConsensusClusterPlus” package.

**S1 B)** Characteristics of the two cluster were presented with heatmap. The cluster 2 integrated HCC samples with relatively higher mir-767-105 cluster expression.

**S1 C)** Kaplan-Meier survival analysis showed that the cluster 2 with relatively higher mir-767-105 cluster expression achieved poorer outcome.

**Fig.S3**


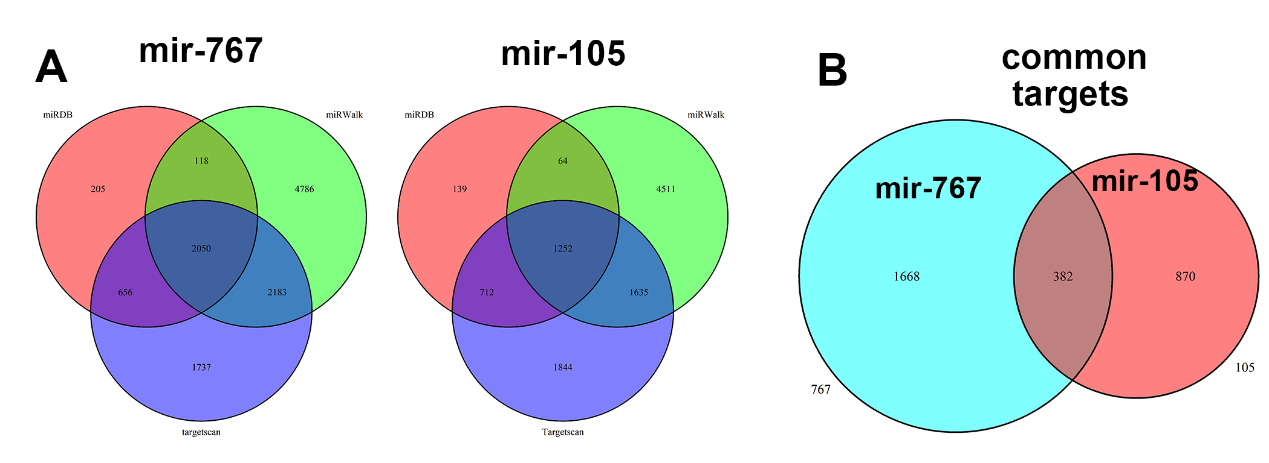


**S A)** The predicted target genes of mir-767 and mir-105 from the miRDB(red colour), miRWalk(green colour), TargetScan (blue colour) databases were overlapped.

**S B)** The mir-767 (green colour) and mir-105 (red colour) target genes were further overlapped. A total of 378 genes may be co-targeted by the cluster.

**Fig.S4**


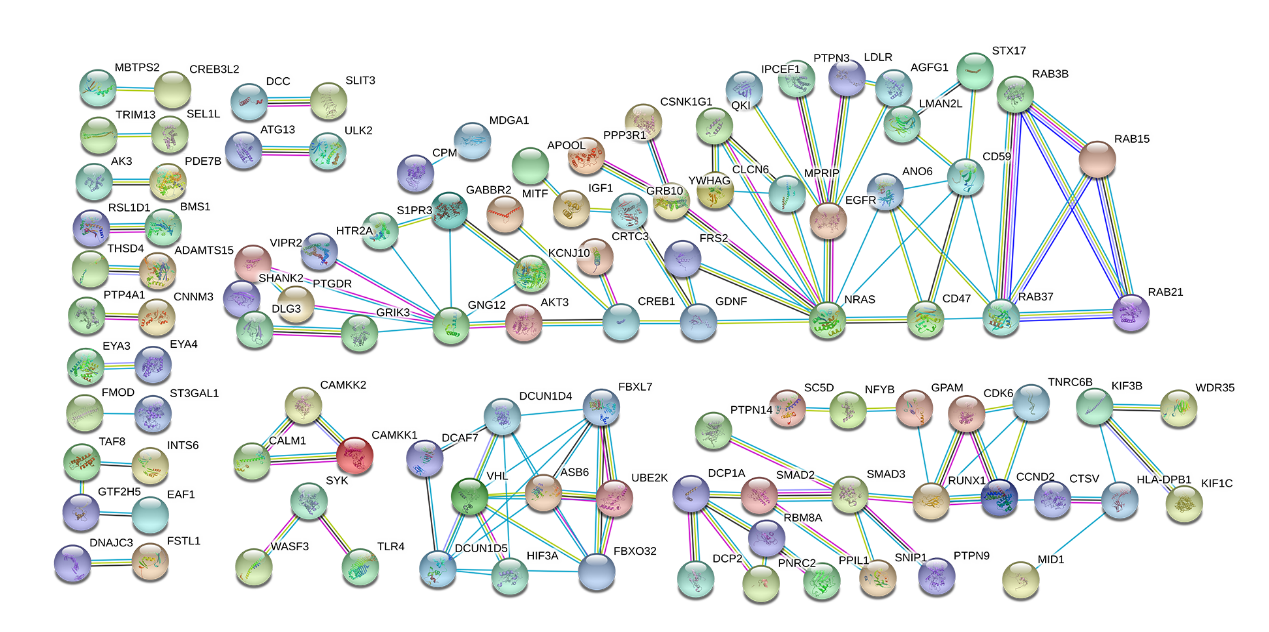


**S3)** Protein-protein interaction analysis showed the target nodes with the highest confidence.
